# Biosynthesis of Promatrix Metalloproteinase-9/Chondroitin Sulphate Proteoglycan Heteromer Involves a Rottlerin-Sensitive Pathway

**DOI:** 10.1371/journal.pone.0020616

**Published:** 2011-06-01

**Authors:** Nabin Malla, Eli Berg, Ugo Moens, Lars Uhlin-Hansen, Jan-Olof Winberg

**Affiliations:** Department of Medical Biology, Faculty of Health Sciences, University of Tromsø, Tromsø, Norway; University of Bergen, Norway

## Abstract

**Background:**

Previously we have shown that a fraction of the matrix metalloproteinase-9 (MMP-9) synthesized by the macrophage cell line THP-1 was bound to a chondroitin sulphate proteoglycan (CSPG) core protein as a reduction sensitive heteromer. Several biochemical properties of the enzyme were changed when it was bound to the CSPG.

**Methodology/Principal Findings:**

By use of affinity chromatography, zymography, and radioactive labelling, various macrophage stimulators were tested for their effect on the synthesis of the proMMP-9/CSPG heteromer and its components by THP-1 cells. Of the stimulators, only PMA largely increased the biosynthesis of the heteromer. As PMA is an activator of PKC, we determined which PKC isoenzymes were expressed by performing RT-PCR and Western Blotting. Subsequently specific inhibitors were used to investigate their involvement in the biosynthesis of the heteromer. Of the inhibitors, only Rottlerin repressed the biosynthesis of proMMP-9/CSPG and its two components. Much lower concentrations of Rottlerin were needed to reduce the amount of CSPG than what was needed to repress the synthesis of the heteromer and MMP-9. Furthermore, Rottlerin caused a minor reduction in the activation of the PKC isoenzymes δ, ε, θ and υ (PKD3) in both control and PMA exposed cells.

**Conclusions/Significance:**

The biosynthesis of the proMMP-9/CSPG heteromer and proMMP-9 in THP-1 cells involves a Rottlerin-sensitive pathway that is different from the Rottlerin sensitive pathway involved in the CSPG biosynthesis. MMP-9 and CSPGs are known to be involved in various physiological and pathological processes. Formation of complexes may influence both the specificity and localization of the enzyme. Therefore, knowledge about biosynthetic pathways and factors involved in the formation of the MMP-9/CSPG heteromer may contribute to insight in the heteromers biological function as well as pointing to future targets for therapeutic agents.

## Introduction

Proteoglycans (PGs) constitute an own entity of glycoproteins, where the core proteins are substituted with glycosaminoglycan (GAG) chains. There are several types of GAG-chains, where chondroitin sulphate (CS) is one of the major types. CS-chains are unbranched and contain a variable number of negatively charged sulphate groups which are important for their function [1], [2]. Almost all mammalian cells synthesize PGs, and these are either secreted or cell associated. PGs synthesized from monocytes and macrophages are mainly substituted with CS-chains (CSPG) [3]–[7]. When monocytes are stimulated and differentiated to macrophages, both the biosynthesis and the secretion of CSPG are increased [7]. The human monocyte cell line THP-1 secretes PGs such as serglycin, versican and perlecan [8], [9]. The biological role of the secreted PGs such as serglycin from macrophages is not clear, but it has been shown that they bind to other molecules released from the cells through interaction with the GAG-chains, suggesting that serglycin and other PGs may act as a kind of carrier molecule [10], [11]. It has also been reported that serglycin is constitutively produced by multiple myeloma plasma cells and can inhibit the bone mineralization process [12].

The family of matrix metalloproteinases (MMPs) consists of approximately 25 different secreted and membrane-bound mammalian enzymes. They are zinc and calcium dependent, and together the MMPs are able to degrade most extracellular matrix (ECM) proteins. In addition they can process and regulate the activity of a large amount of non-ECM proteins such as growth factors, cytokines, chemokines, cell receptors, serine proteinase inhibitors as well as other MMPs [13]–[17]. Thus, MMPs have complicated biological functions playing a role in both normal and pathological conditions [15], [18]–[20].

All MMPs are built up of various modules, including a pro- and a catalytic domain. In addition, all the secreted MMPs with the exception of the two matrilysins (MMP-7/-26) also contain a C-terminal hemopexin-like domain [15], [16]. Secreted MMPs bind to ECM proteins, PGs as well as cell membranes [21]. The two gelatinases MMP-2 and MMP-9 contain a unique inserted domain in their catalytic region, i.e. a module containing three fibronectin II-like repeats (FnII). This domain is similar but not identical in the two gelatinases, and is involved in the binding of denatured collagens, elastin and native collagen. The three FnII-like repeats in the catalytic site of MMP-2 and MMP-9 may facilitate the localization of these enzymes to connective tissue matrices. They also appear to be of importance for the degradation of macromolecules such as elastin, gelatin and collagens IV, V and XI, but do not influence the degradation of chromogenic substrates [22]–[31].

MMP-9 (92 kDa gelatinase) is produced by a variety of cell lines, including monocytes and macrophages. MMP-9 is produced as a monomer as well as various dimer forms [32]–[38]. The homo- and several of the hetero-dimer forms are reduction sensitive. Hence, the proteins are either covalently linked to each other through disulfide bonds or a very strong reversible interaction where intramolecular disulfide bonds are essential. We discovered that THP-1 cells produced a new type of reduction sensitive heteromer, where 10–15% of the total amount of proMMP-9 synthesized was linked to the core protein of one or several CSPGs [38]. The binding of proMMP-9 to a CSPG alters several biochemical properties of the enzyme [39], [40]. Among these properties are the ability to be activated by organomercurial compounds as well as its binding to collagens and gelatins through the three FnII repeats in the enzymes catalytic region. It was shown that the CSPG core protein hides and prevents binding of collagen and gelatin to the enzymes FnII domain.

The aim of the present investigation was to determine whether different compounds known to stimulate the synthesis of proMMP-9 and CSPGs also can increase the syntheses of proMMP-9/CSPG heteromers in THP-1 cells, and to what extent various PKCs are involved. Our results revealed that only PMA significantly increased the biosynthesis of the heteromers and that a Rottlerin sensitive pathway is involved.

## Materials and Methods

### Materials

Formaldehyde, TRIS, urea, DMSO and sodium acetate were from Merck. Trichloroacetic acid (TCA), Ethanolamine and EDTA were from Fluka. Acrylamide, Coomassie Brilliant Blue G-250 and Triton X-100 were from BDH. Safranin O (no.S-2255), cetylpyridinuim chloride, phorbol 12-myristate 13-acetate (PMA), blue dextran, Hepes, Brij-35, alkaline phosphatase-conjugated antibodies, chondroitin sulphate C, gelatin, bovine serum albumine (BSA), LPS, Concanavalin A (ConA), Sigma Fast Protease inhibitor cocktail tablets, primers and the JumpStart™Red Taq® PCR reaction mix were purchased from Sigma. IL-1α and IL-1β were from R&D Systems, while PGE_2_ (prostaglandin E_2_) was from Cayman Chemicals. Proteinase free chondroitin ABC lyase (cABC) was from Seikagaku Kogyo Co. Gelatin-Sepharose, Q-Sepharose, Superose 6 (HR 10/30), Sephadex G-50 (fine), Amplify and ^14^C-labeled Rainbow™ protein molecular weight standards were from GE-Healthcare. Ultima Gold XR, [^35^S]sulphate and [^3^H]glucosamine were obtained from Perkin Elmer. DC Protein Assay and unlabeled molecular weight standards were from BioRad. Magic Marker molecular weight standards and pre-casted polyacrylamide gels (NuPAGE Novex 4–12% Bis-Tris gels) for Western blotting were from Invitrogen. Solution cell proliferation assay (Cell Titer 96AQueous One) was from Promega. Rabbit polyclonal antibody against human PKCα, rabbit monoclonal antibodies against human Phospho-MAPKAPK-2 (Thr222) and PKCε were from Cell Signaling Technology. Western Blotting Luminol Reagent, HRP-conjugated donkey anti-goat secondary antibody, rabbit polyclonal antibodies against human ERK2, PKC βI, βII, δ and goat polyclonal antibody against human PKCθ were from Santa Cruz Biotechnology. Rabbit polyclonal antibody against PKD3 was a kind gift from Dr. Johan Van Lint, Catholic University, Leuven, Belgium. HRP-conjugated goat anti-rabbit secondary antibody was from Southern Biotech. MCSF (macrophage colony stimulating factor), TNF-α, bFGF, IL-3, IL-6, Gö6983, Gö6976 and Rottlerin were from Calbiochem. NucleoSpin RNAII kit was from Macherey-Nagel. iScript cDNA synthesis kit and DC Protein assay kit was from Bio-Rad. Kodak Scientific imaging film, X-OMAT AR was from Kodak.

### Biosynthesis of proMMP-9/CSPG heteromer, proMMP-9 and CSPGs

The human leukemic monocyte cell-line THP-1 was a kind gift from Dr. K. Nilsson, Department of Pathology, Uppsala University, Sweden. The cells were cultured in RPMI 1640 medium with 10% fetal bovine serum, 50 µg/ml of streptomycin, and 100 units/ml of penicillin. To investigate the effect of various biological compounds on the biosynthesis of proMMP-9/CSPG heteromer, proMMP-9 and CSPGs, cells were washed 3 times in serum-free medium and then cultured for 72 h in serum-free RPMI 1640 medium with or without the following compounds; PMA (10^−9^–10^−5^ M); IL-1α (1–200 ng/ml), IL-1β (1–200 ng/ml), IL-3 (1–200 ng/ml), IL-6 (1–200 ng/ml), LPS (1–5000 ng/ml), ConA (1–200 µM; 26–5200 µg/ml), TNF-α (1–200 ng/ml), bFGF (1–200 ng/ml), PGE_2_ (1–200 ng/ml), MCSF (1–200 ng/ml). In all cases, control cells contained the same amount of DMSO as the cells treated with the various biological compounds. Conditioned medium was harvested and loose cells were removed by centrifugation at 1000 rpm for 10 min. CSPG and proMMP-9/CSPG heteromers were thereafter isolated and detected as described below. To determine proMMP-9 the enzyme was detected directly in the harvested medium by gelatin zymography.

### Preparation of cytosol and plasma membranes

Sixty million cells treated with or without PMA (10^−7^ M) and Rottlerin (5 µM) were incubated in serum-free RPMI 1640 medium for 8 hr as described above. Adherent cells (PMA treated) were scraped off and suspended in PBS. Cells were thereafter pelleted for 10 min at 2000 rpm and re-suspended in 50 mM Tris-HCl buffer pH 8.0 containing 5 mM CaCl_2_ and 1x proteinase inhibitor cocktail along with 10 mM EDTA. Cells were homogenized with a glass homogenizer and the cytosol fraction was separated from plasma membrane, intact cells and organelles by centrifugation (50,000xg for 1 h). The supernatant fraction (cytosol) was collected and the pellet was dissolved in 20 mM Tris-HCl buffer pH 7.4 containing 8.7% sucrose, 1x proteinase inhibitor cocktail along with 10 mM EDTA and thereafter layered on the top of a 38.5% sucrose cushion prior to centrifugation (100,000xg, 1 h). The plasma membrane fraction was collected from the interface, pelleted by centrifugation (100,000xg, 1 h), and re-suspended in 50 mM Tris-HCl buffer pH 8.0 containing 5 mM CaCl_2_, 1x proteinase inhibitor cocktail along with 10 mM EDTA [41], [42]. All steps were performed at 4°C. The cytosol and plasma membrane fractions were frozen and stored at −70°C. The amount of protein in the cytosol and membrane fractions was determined by the DC Protein assay kit using BSA as a standard. Four independent measurements were performed on each cytosol and membrane preparation, and the standard deviation of the determined protein concentrations was less than 10%.

### RNA isolation and reverse transcriptase-polymerase chain reaction (RT-PCR)

Total RNA was isolated using the NucleoSpin RNAII kit according to the manufacturer's protocol. Two µg of RNA was reverse-transcribed using iScript cDNA synthesis kit. The primers for PCR were: PKCα forward: 5′-atccgcagtggaatgagtcctttacat-3′; PKCα reverse: 5′-ttggaaggttgtttcctgtcttcagag-3′; PKCβI forward: 5′-ctgtggaactgactcccactg-3′; PKCβI reverse: 5′-atactgaagcattttggtatc-3′; PKCβII forward: 5′-gaccgatttttcacccgcca-3′; PKCβII reverse: 5′-ccatctcatagagatgctcc-3′; PKCγ forward: 5′-cacgaagtcaagagccacaa-3′; PKCγ reverse: 5′-tagctatgcaggcggaactt-3′; PKCδ forward: 5′-caactacatgagccccacct-3′; PKCδ reverse: 5′-gaggctctctgggtgacttg-3′; PKCε forward: 5′-gatgcagaaggtcactgcaa-3′; PKCε reverse: 5′-gtcgtcatggaggatggact-3′; PKCξ forward: 5′-gttatcgatgggatggatgg-3′; PKCξ reverse: 5′-gcaccagctctttcttcacc-3′; PKCη forward: 5′-gaacagaggttcgggatcaa-3′; PKCη reverse: 5′-atatttccgggttggagacc-3′; PKCθ forward: 5′-acaaacagggctaccagtgc-3′; PKCθ reverse: 5′-atgccacatgcatcacactt-3′; PKCι forward: 5′-tacggccaggagatacaacc-3′; PKCι reverse: 5′-tcggagctcccaacaatatc-3′; PKCµ/PKD1 forward: 5′-acggcactattggagattgg-3′; PKCµ/PKD1 reverse: 5′-tgaccacattttctcccaca-3′; PKD2 forward: 5′-gccctcacggtgcactcctatc-3′; PKD2 reverse: 5′-tgacaccggagtcctctgact-3′; PKCυ/PKD3 forward: 5′-atttgtctacaagtatctctgt-3′; PKCυ/PKD3 reverse: 5′-ctgaccacatatctagggaacg-3′. The PCR cycling conditions were 35 times at 94°C for 30 sec, 55–60°C for 30 sec and 72°C for 30 sec. PCR was performed using JumpStart™Red Taq® PCR reaction mix. PCR products were visualized on an ethidium bromide-stained agarose gel.

### Cell Toxicity and Viability Assays

Cell viability tests were performed in 96-well plates, where 6×10^4^ cells/well were incubated with increasing concentrations of different cytokines, growth factors, lectins, lipopolysaccharides, PMA and PKC inhibitors in 100 µl serum-free RPMI 1640 media for 8 to 72 h. At the end of incubation, cells were washed in PBS and then 100 µl serum-free RPMI 1640 media containing 20 µL of 3-(4,5-dimethythizol-2-yl)-5-(3-carboxymethoxyphenyl)-2-(4-sulfophenyl)-2H-tetrazolium salt (CellTiter 96 AQueous One Solution) was added to each well and further incubated for 1 or 4 h at 37°C and then the absorbance at 490 nm was determined in a plate reader (VersaMax™, Molecular Devices Corp) according to the manufacturer's protocol. Absorbance of control cultures (which was not significantly altered after 72 h of incubation in serum-free conditions) was defined as 100% and compared with that of treated cultures. It was also determined to which extent PMA and the PKC inhibitor Rottlerin were toxic to the cells by using the Trypan Blue test. Briefly, 4×10^5^ cells were added to each well in 12 well plates (0.6 ml/well) and incubated for 12–72 h in the presence or absence of different concentrations of PMA and Rottlerin. Cells were thereafter harvested, mixed with Trypan Blue and counted in a Bürker chamber. To harvest PMA treated cells, a rubber scraper was used to detach the cells from the well.

### Biosynthetic radiolabelling of GAG-chains

To determine the effect of the various biological compounds on the biosynthesis of CSPG, radioactive labelling was performed by incubating 0.8×10^6^ cells in 1 ml with [^35^S]sulphate (50 µCi/ml) for 72 h. To determine the effect of Rottlerin in the presence and absence of PMA (10^−7^ M), GAG-chains were labelled with either 50 µCi/ml of [^35^S]sulphate or 10 µCi/ml of [^3^H]glucosamine by incubating 1×10^6^ THP-1 cells for 8 and 23 h in 0.5 ml of serum-free RPMI 1640 medium containing 50 µg/ml of streptomycin and 100 units/ml of penicillin. Conditioned medium and cells were thereafter separated through centrifugation at 2000 rpm for 10 min. The cell-free conditioned medium and the obtained cell lysates were then applied to a Sephadex G-50 column in order to separate free [^35^S]sulphate or free [^3^H]glucosamine from CSPG-labelled with [^35^S]sulphate or [^3^H]glucosamine. The radioactively labelled CSPG in the media or in the purified material was thereafter counted in a liquid scintillation spectrometer or applied to SDS-PAGE followed by autoradiography.

### Detection of PG-bound CS-chains

PG-bound CS-chains were quantified spectrophotometrically by the Safranin O method as described previously [38].

### Isolation of secreted CSPG and proMMP-9/CSPG heteromers

Secreted PG and the proMMP-9/PG heteromers were isolated by Q-Sepharose anion-exchange chromatography as described previously [38].

### Isolation and detection of radioactive labelled CSPG and free CS chains

To determine the size of CSPGs isolated from Rottlerin-treated and untreated cells, as well as CS-chains released from CSPGs, Q-Sepharose purified [^35^S]sulphate and [^3^H]glucosamine labelled CSPG were applied to a Superose 6 (HR19/30) column. The latter column was run in 50 mM sodium acetate, 4M guanidine and 0.15 M NaCl, pH 6.0 and markers for void (V_0_) and total volume (V_t_) were blue dextran and free [^35^S]sulphate, respectively. Furthermore, to determine if a size difference between the CSPGs isolated from Rottlerin-treated and untreated cells could be detected on SDS-PAGE, ^35^S-labelled CSPG (30000 cpm/well) was subjected to SDS-PAGE using either a 4–12% gradient gel or a 7.5% gel (as in the zymography experiments but without added gelatin). After the electrophoresis, the gel was soaked in Amplify and dried and [^35^S]CSPG was detected with autoradiography.

### Purification of proMMP-9 from the THP-1 cells

The proMMP-9 in conditioned medium from the THP-1 cells was partly purified as described previously [39].

### Chondroitin ABC lyase (cABC) treatment

The PG bound CS-chains were removed by digestion for 2 h at 37°C with 0.2–1.0 units of cABC/ml in 0.05 M Tris-HCl, pH 8.0, containing 0.05 M sodium acetate.

### Gelatin zymography

SDS-substrate PAGE was done as described previously [38] with gels (7.5 cm × 8.5 cm × 0.75 mm) containing 0.1% (w/v) gelatin in both the stacking and separating gel, 4 and 7.5% (w/v) of polyacrylamide, respectively. Gelatinase activity was evident as cleared (unstained) regions, and the area of these regions was quantified by the GelBase/GelBlot™ Pro computer program from Ultra Violet Products.

### Western immunoblotting analysis of PKC and PKD isoforms

Immunoreactive PKC and PKD isoforms were determined by immunoblotting of cytosol and plasma membrane preparations subjected to SDS-PAGE (NuPAGE Novex 4–12% Bis-Tris gels) before electroblotting to a polyvinyl difluoride (PVDF) membrane. Cytosolic and plasma membrane preparations were obtained as described above and a given amount of protein was loaded to the gels. After blockage of non-specific binding sites with non-fat milk in TBS-T (150 mM NaCl, 0.25% Tween-20, 20 mM Tris-HCL, pH 7.4) for 1 h at room temperature, a specified amount of cytosol and plasma membrane were incubated with antibodies against the PKC and PKD isoforms over night at 4°C. One blot for each PKC isoenzyme was also incubated with an antibody against ERK2 as an additional loading control. ERK2 has been shown to be unaffected by PMA and rottlerin [43], [44] and is located both in the cytosol and at the plasma membrane [45]. After the incubation with primary antibodies, the PVDF membranes were washed in TBS-T 3×5 min, and incubated with HRP-conjugated donkey anti-goat secondary antibody or goat anti-rabbit secondary antibody for 1 h and washed 3×5 min with TBS-T before visualization using a Western Blotting Luminol Reagent. The intensity of immunoblot bands was measured using a Luminescent Image Analyzer LAS-3000 with MultiGauge software version 3.0 (Fujifilm, Tokyo, Japan).

### Statistical analysis

In all cases, at least three independent cell experiments were performed. Assays on all harvested media and cell fractions were performed in triplicate or more (unless otherwise stated) with data presented as mean ± S.D., using the Student *t* test.

## Results

### Cell morphology and viability

Untreated THP-1 cells cultured on plastic had a spherical morphology and were non-adherent. When the cells were exposed to the tumour promoting phorbol ester PMA they became adherent, more flattened and spread with pseudopodia. The other compounds used did not induce the same morphological changes. Similar observations for PMA treated THP-1 cells were previously described by Van Ranst and coworkers [46].

**Figure 1:**
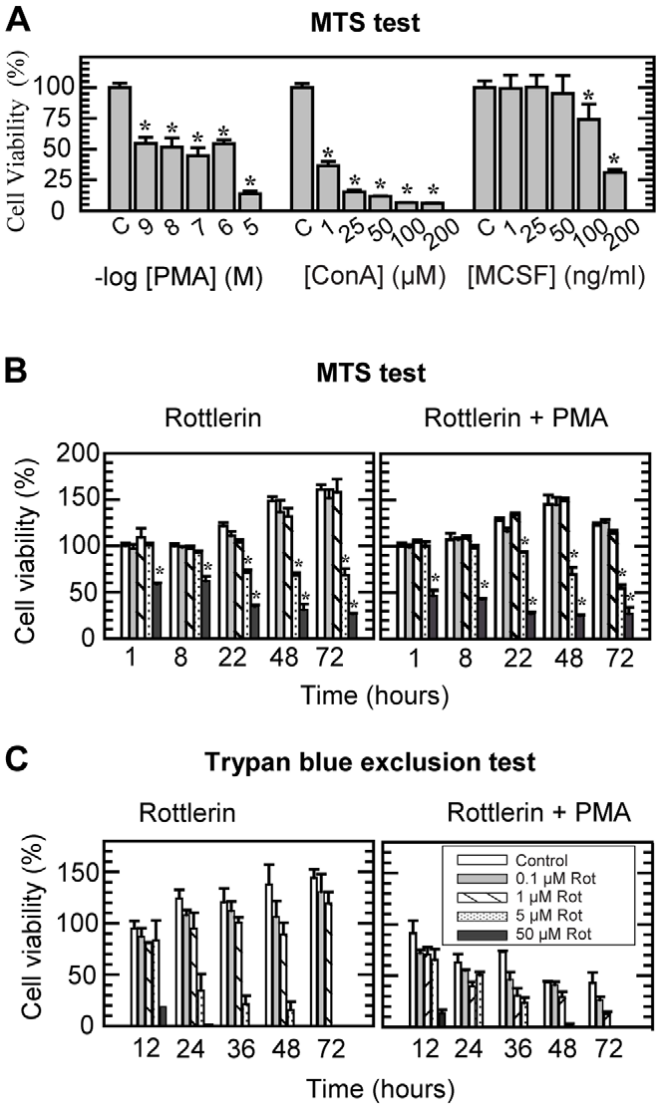
Cell viability in the presence of PMA, ConA, MCSF and Rottlerin. Cell viability was detected by the MTS assay (**A, B**) and by counting viable cells in a Bürker chamber (**C**) using the Trypan Blue exclusion test. In (**A**) 6×10^4^ cells in serum free medium were seeded per well in 96 well plates in the absence or presence of PMA, ConA or MCSF at the indicated concentrations, and incubated for 72 h. In (**B**) and (**C**) the cells were incubated with various concentrations of Rottlerin (as indicated in the insert in c) either in the absence or presence of 10^−7^ M PMA. In (**B**) 6×10^4^ cells were seeded per well in 96 well plates and in (**C**) 4×10^5^ cells were added to each well in 12 well plates (0.6 ml/well) and incubated in serum free medium for the indicated time period. The results presented are from a typical experiment where N = 4 in (A), 3 in (B) and 2 in (C); *p<0.05 compared to control.

Prior to examining the effect of the various compounds on the THP-1 cells synthesis of proMMP-9/CSPG heteromer, proMMP-9 and CSPG, we determined to which extent these compounds affected the cell viability. For this purpose two different methods were used, the MTS and Trypan Blue exclusion test. At the concentrations used (see methods), none of the following compounds (IL-1α, IL-1β, IL-3, IL-6, PGE_2_, TNF-α, bFGF and LPS) had any effect on the viability based on the MTS test. In contrast to this, a decrease in viability was observed when the cells were exposed to MCSF, ConA and PMA (Fig. 1A). In the case of PMA, the Trypan Blue exclusion test verified that PMA was slightly toxic to the cells. Four independent experiments where the THP-1 cells were cultured in the absence or presence of 10^−7^ M PMA for 24, 48 and 72 h resulted in 69±12, 55±12 and 56±14% (mean ± SD) of living cells in PMA exposed cultures. The percentage of dead cells (mean ± SD from four independent experiments) after 24, 48 and 72 h were 5±2, 6±3 and 8±5 in control cultures and 31±5, 45±13 and 44±6 in PMA exposed cultures.

The two PKC inhibitors Gö6976 (25 nM) and Gö6983 (25 and 100 nM) had no effect on the cell viability neither in the absence or presence of 10^−7^ M PMA using the MTS test (data not shown). However, time- and dose-dependent studies with the PKC inhibitor Rottlerin in the absence or presence of PMA showed that Rottlerin was toxic for the cells as monitored by the MTS and the Trypan blue exclusion tests (Figs. 1B and C)

### PMA stimulates the biosynthesis of the proMMP-9/CSPG heteromer in a time and concentration dependent manner

**Figure 2:**
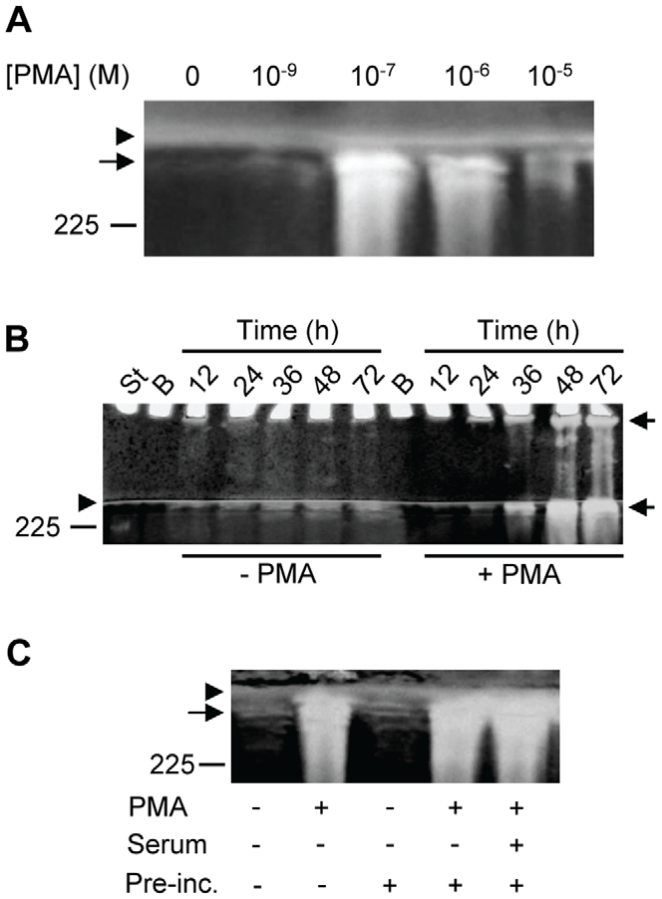
Synthesis of proMMP-9/CSPG in the absence and presence of PMA. THP-1 cells were incubated in the absence or presence of PMA in serum free medium. Harvested medium was thereafter applied to Q-Sepharose chromatography and the presence of proMMP-9/CSPG was detected with gelatin zymography as described in Materials and Methods. In (**A**), cells were incubated for 72 h in the presence of various concentrations of PMA as indicated. In (**B**), cells were incubated for various time periods (as indicated) in the absence or presence of 10^−7^ M PMA. The samples (containing CSPG and proMMP-9/CSPG complex) from cells not exposed to PMA were five times more concentrated than the samples from the PMA treated cells when applied to the gel. In (**C**), cells were either incubated for 72 h in the absence (−) or presence (+) of 10^−7^ M PMA at serum free conditions, or pre-incubated for 3 h in the absence (−) or presence (+) of 10^−7^ M PMA and/or 10% fetal calf serum. After the pre-incubation, cells were washed three times in PBS and thereafter incubated in serum free medium for 72 h. Arrowhead shows the border between the separating and stacking gel, and arrows show the position of the proMMP-9/CSPG complexes. Purified proMMP-9 was used as a standard and the position of the 225 kDa homodimer form is shown at the left. The gels are representative for several similar experiments.

PMA is known to stimulate monocytic cell lines to increase the synthesis of MMPs [46]–[49]. We therefore determined to what extent PMA could stimulate the biosynthesis of the proMMP-9/CSPG heteromer by THP-1 cells. In several independent experiments the biosynthesis of the proMMP-9/CSPG heteromers increased with increasing PMA concentrations with a peak at 10^−7^ M, and thereafter decreases with increasing PMA concentrations (Fig. 2A).

To study the time dependency of the accumulation of the proMMP-9/CSPG heteromer in the culture medium, cells were cultured in the absence or presence of PMA (10^−7^ M) for 8 to 72 h before the amount of proMMP-9/CSPG heteromers was determined as described in Materials and Methods. Figure 2B shows that the heteromer can be detected already after 12 h from both untreated and PMA-treated cells, and that the amount of heteromer increased with time for at least 72 h. In other experiments with more material loaded to the gelatin zymography gel, it was shown that the complex could be detected already after 8 h from both untreated and PMA treated cells (data not shown).

As it was shown that the proMMP-9/CSPG heteromer accumulated with time for at least 72 h in the PMA exposed cells, the question arise whether it is necessary with a continuous exposure to the phorbol ester. Cells were therefore primed with PMA for 3 h prior to the incubation for 72 h in the absence of PMA. In control cultures, PMA was present during the entire 72 h period. The cells produced comparable amounts of the heteromer whether the PMA was removed after 3 h or not (Fig. 2C). Likewise, whether pre-incubation of the cells with PMA occurred in serum or serum-free media had no detectable effect on the biosynthesis of the heteromer (Fig. 2C).

### PMA stimulation affected the biosynthesis of the two components of the proMMP-9/CSPG heteromer differently

**Figure 3:**
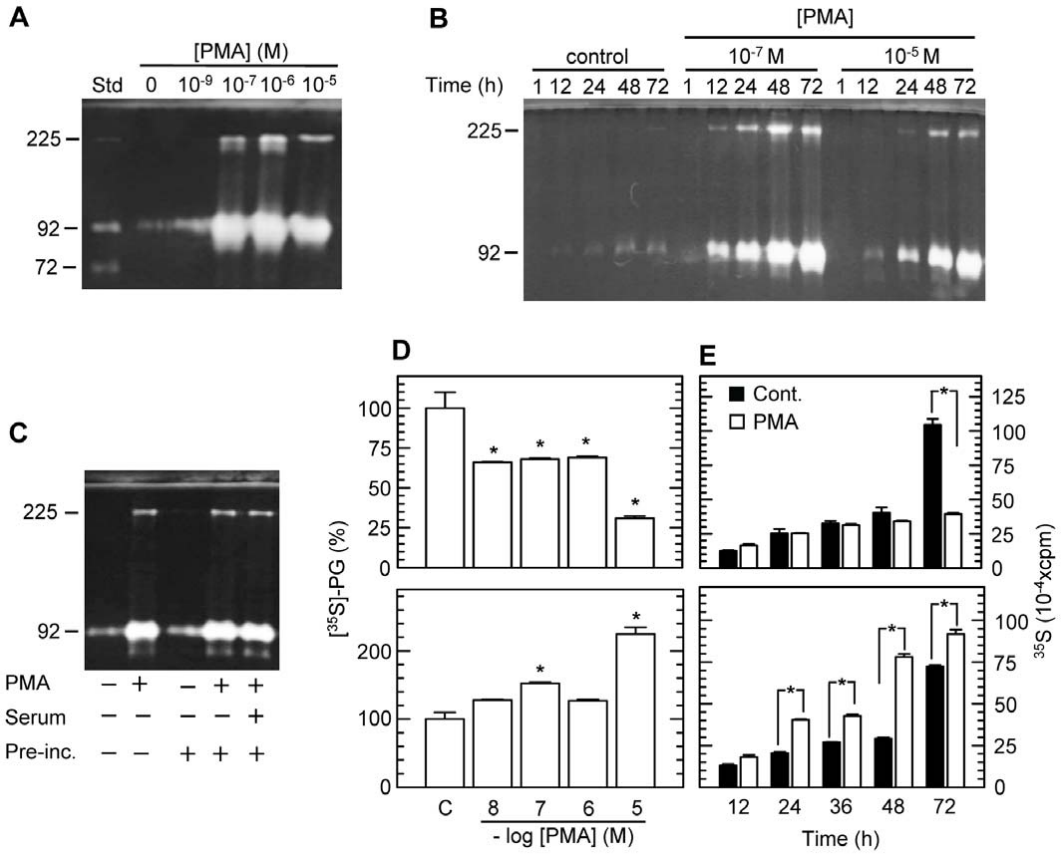
Synthesis of proMMP-9 and CSPG in the absence and presence of PMA. **A**–**C**: Typical gelatin zymographies of 20 times diluted conditioned medium from THP-1 cells. **A**: Cells were incubated for 72 h in the presence of various concentrations of PMA as indicated. **B**: Cells were incubated for various time periods (as indicated) in the absence or presence of 10^−7^ M and 10^−5^ M PMA. **C**: Cells were either incubated for 72 h in the absence (−) or presence (+) of 10^−7^ M PMA at serum free conditions, or pre-incubated for 3 h in the absence (−) or presence (+) of 10^−7^ M PMA and/or 10% fetal calf serum. After the pre-incubation, cells were washed three times in PBS and thereafter incubated in serum free medium for 72 h. **A–C**: The position of proMMP-9 homodimer (225 kDa), monomer (92 kDa) and proMMP-2 (72 kDa) standards is indicated. **D** and **E**: Conditioned medium from THP-1 cells incubated with [^35^S]sulphate was passed over a G-50 Sepharose column in order to separate labelled macromolecules from free [^35^S]sulphate. The amount of labelled macromolecules was determined by counting the entire pass through fraction in a liquid scintillation spectrometer. **D**: Cells were incubated in the absence or presence of various concentrations of PMA for 72 h as indicated and in (**E**) the cells were incubated for various time periods in the absence or presence of 10^−7^ M PMA. (**D**: upper and lower panel) Results (mean ± s.d) were normalized against the control (without PMA). Lower panel, results were in addition normalized against the number of viable cells. **E**: Results (mean ± s.d) are presented as cpm (upper panel) and cpm/viable cells (lower panel). The results in (**D**) and (**E**) are from a typical experiment with four parallels in (D) and three parallels in (E), where *p<0.05 compared to control without PMA.

PMA stimulation of the THP-1 cells resulted in a concentration (Fig. 3A) and time (Fig. 3B) dependent increase of proMMP-9 in the cell conditioned media. Likewise, the PMA induced synthesis of proMMP-9 continued after the removal of the phorbol ester (Fig. 3C). Whether pre-incubation of the cells with PMA occurred in serum or serum-free media had no detectable effect on the biosynthesis of the proMMP-9 monomer and homodimer (Fig. 3C).

In contrast to the PMA-induced increase in proMMP-9 and proMMP-9/CSPG synthesis, PMA reduced the amount of [^35^S]CSPG secreted into the medium in a concentration dependent manner (Fig. 3D, upper panel). However when the amount of secreted [^35^S]CSPG was normalized against the number of viable cells, a slight increase was observed in the PMA exposed cell cultures (Fig. 3D, lower panel). In both untreated and PMA treated cells, approximately 90% of the CSPG synthesized was secreted into the culture medium. The amount of CSPG in the culture medium increased with time in both the untreated and PMA treated cells. After 72 h the conditioned culture medium from control cells contained approximately 2.5 times more [^35^S]CSPG than the culture medium from PMA treated cells (Fig. 3E, upper panel). However when the amount of CSPG was normalized against the number of viable cells, a slight increase in [^35^S]CSPG was found in the culture medium from PMA treated cells at all time points (Fig. 3E, lower panel).

### Untreated and PMA treated THP-1 cells express various PKC mRNA isoforms

**Figure 4:**
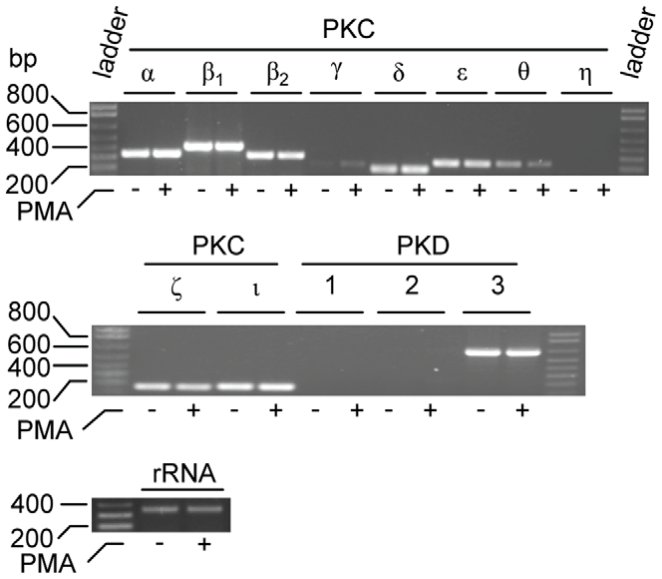
PKC and PKD isoenzymes expressed in THP-1 cells. RT-PCR was used to detect the PKC and PKD isoenzyme mRNA present in cells incubated in the absence (−) or presence (+) of 10^-7^ M PMA. To ensure that equal amounts of cDNA has been used in samples from control and PMA stimulated cells, rRNA was used as a standard. The probes used are described in Materials and Methods. Ladder is a marker with DNA fragments of known size (in bp).

Previously it has been shown that undifferentiated THP-1 cells express the classical PKC isoforms α and β, novel PKC isoforms δ and ε and the atypical PKC ζ [50]. The same authors showed that PMA changed the expression of some of these PKC isoforms. As PMA is known to induce activation of various classical and novel PKC isoforms [51], we have reinvestigated at the mRNA level which isoforms are produced by THP-1 cells in the presence and absence of PMA. This has extended the list of PKC isoforms present in these cells. As shown in Figure 4, both untreated and PMA treated THP-1 cells contained mRNA for classical PKC isoforms α, βI and βII, novel PKC isoforms δ, ε and θ, atypical PKC ζ and ι as well as for PKD3 (PKCυ). The signals for PKC isoforms θ and γ were weak, and no mRNA could be detected for the PKC η, and the PKD isoforms 1 (PKC μ) and 2 (Fig. 4). As also shown in Figure 4, exposure to PMA did not seem to affect the mRNA levels of the PKC isoforms in THP-1 cells.

### Rottlerin blocks the biosynthesis of proMMP-9/CSPG, proMMP-9 and CSPG

**Figure 5:**
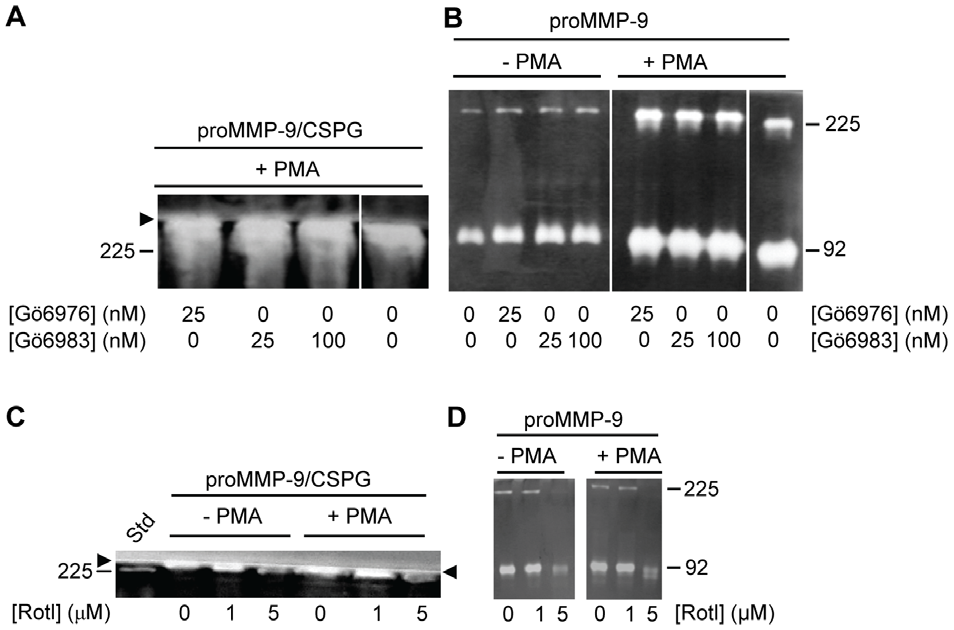
Effect of PKC inhibitors on THP-1 cells synthesis of proMMP-9/CSPG heteromer and proMMP-9. Shown is a typical experiment where cells in the absence (−) and presence (+) of PMA (10^−7^ M) were incubated with various concentrations of the PKC inhibitors Gö6976 and Gö6983 (**A, B**) and Rottlerin (**C, D**). In the presence of Gö6976 and Gö6983 (**A, B**), cells were incubated for 72 h, while in the presence of Rottlerin (**C, D**) the cells were incubated for 12 h in serum free medium. To detect the effect of the PKC inhibitors on the synthesis of the proMMP-9/CSPG heteromer (**A, C**), the harvested media was applied to Q-Sepharose chromatography as described in Materials and Methods prior to gelatin zymography. To determine the effect of the inhibitors on the synthesised proMMP-9 (**B, D**), the harvested medium was diluted 20 times and then applied to gelatin zymography. Arrowhead shows the border between the separating and stacking gel and the position of purified proMMP-9 monomer (92 kDa) and proMMP-9 homodimer (225 kDa) used as a standard (Std) is shown.

We tested to what extent inhibitors of various PKC isoforms could block the PMA induced biosynthesis of the proMMP-9/CSPG complex. Neither 25 nM Gö6976 (inhibits PKC α, β1 and μ) nor 25–100 nM Gö6983 (inhibits PKC α, β1, γ, δ and ζ) affected the PMA induced biosynthesis of the proMMP-9/CSPG complex (Fig. 5A). Furthermore, these two PKC inhibitors did not affect the biosynthesis of the proMMP-9 monomer and homodimer (Fig. 5B).

In contrast to this, Rottlerin which is reported to be a specific inhibitor of the novel PKC δ and PKC θ [44], [52], [53] blocked the biosynthesis of both the proMMP-9/CSPG complex and the proMMP-9 monomer (92 kDa) and homodimer (225 kDa). Rottlerin at a concentration of 5 µM blocked the synthesis in both the untreated and PMA treated cells (Fig. 5C and D). Since Rottlerin is toxic when THP-1 cells were exposed to the substance for more than 12 h (Fig. 1C), the culture medium containing proMMP-9/CSPG heteromer and the proMMP-9 monomer/homodimer was recovered after 12 h in these experiments.

**Figure 6:**
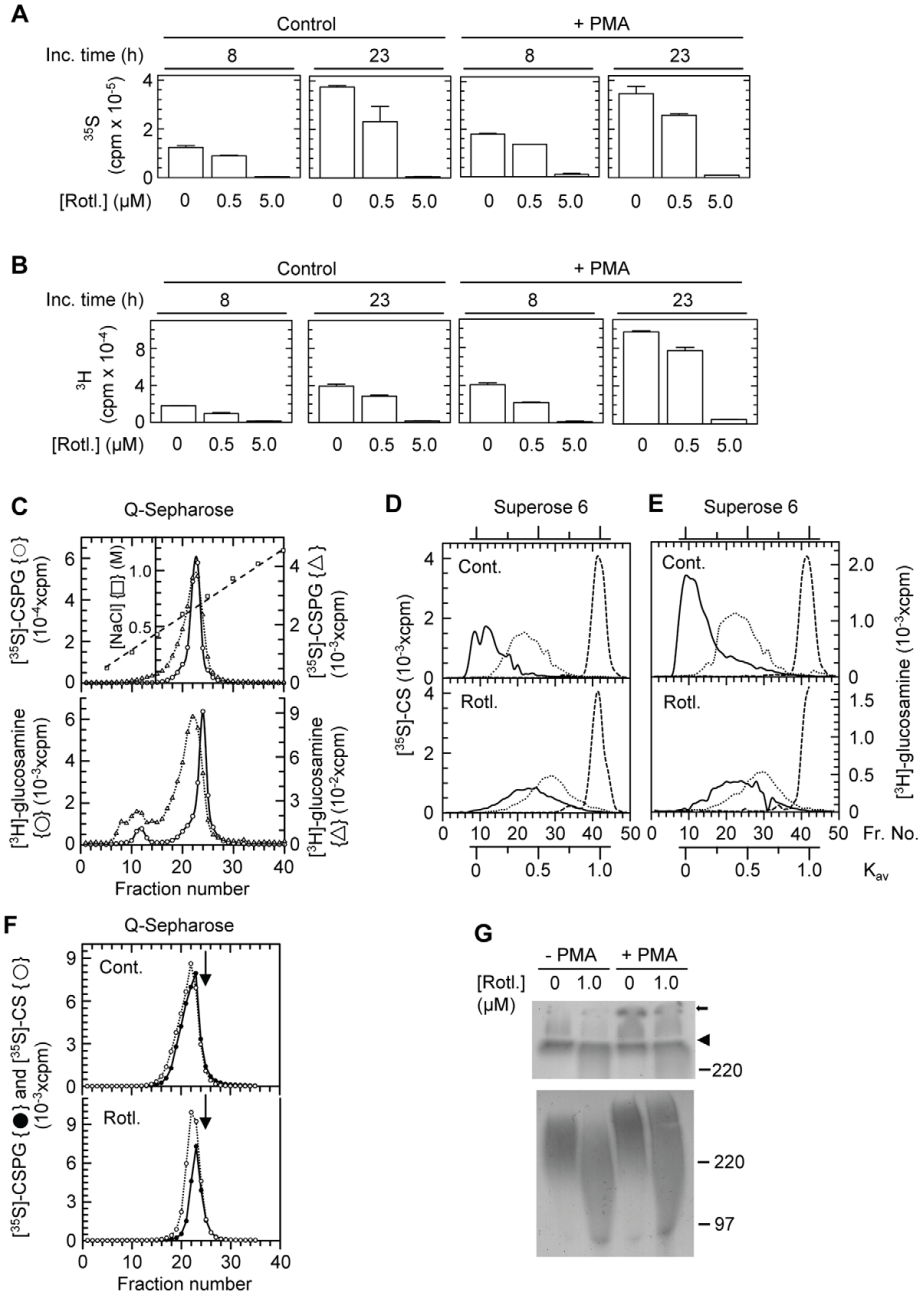
Effect of Rottlerin on biosynthesis and molecular size of CSPG and the CS-chains. **A, B**: Cells in the absence (control) or presence of 10^−7^ M PMA were incubated with increasing concentrations of Rottlerin for 8 h and 23 h in serum free medium containing either [^35^S]sulphate (**A**) or [^3^H]glucosamine (**B**). A typical experiment is shown, where the results (total cpm not adjusted to the amount of living cells) are presented as mean ± s.d. (n = 2). **C**–**F**: Cells were incubated for 24 h in serum free medium containing either [^35^S]sulphate or [^3^H]glucosamine. **C**: [^35^S]sulphate and [^3^H]glucosamine labelled macromolecules were applied to Q-Sepharose chromatography and the bound CSPG were eluted with a 0.15–1.5 M NaCl gradient (----) as shown in the upper graph. ○, absence of Rottlerin; Δ, presence of 1 µM Rottlerin. **D, E**: Eluted CSPG from the Q-Sepharose column was either untreated (solid line) or treated with cABC (---) or 0.5 M NaOH (^…….^) prior to application on a Superose 6 gel chromatography column as described in Material and Methods. Cont., control without Rottlerin; Rotl., presence of 1 µM Rottlerin and Fr.No., fraction number. **F**: [^35^S]CSPG (•) and free [^35^S]CS-chains (○) from control and Rottlerin-treated cells were subjected to Q-Sepharose chromatography as in (C). Arrow shows the elution position of shark cartilage CS. **G**: [^35^S]Sulphate labelled CSPG (isolated from control, PMA (10^−7^ M) and Rottlerin treated cells) was subjected to SDS-PAGE (upper panel: 4% stacking gel and 7.5% separating gel; lower panel: 4–12% gradient gel) followed by autoradiography (see Materials and Methods). An equal amount of radioactivity (based on scintillation counting) was loaded to each well in order to be able to compare the bands. Arrowhead shows the border between the separating and stacking gel and the position of molecular size markers are shown. Small arrow shows the bottom of the application well.

We have previously shown that ∼0.1% of the total amount of CSPGs sythesized by the THP-1 cells are proMMP-9/CSPG heteromers [39]. Therefore, biosynthetic labelling of the CS-chains with either [^35^S]sulphate or [^3^H]glucosamine in the absence or presence of PMA and Rottlerin will reflect the latter compounds effect on the cells biosynthesis of CSPGs. The results revealed that the biosynthesis of the second component of the complex, the CSPG, was also inhibited by Rottlerin in a concentration dependent manner. As shown in figures 6(A) and 6(B), the conditioned culture medium from cells exposed to Rottlerin contained less amounts of the [^35^S]sulphate and [^3^H]glucosamine labelled macromolecules than medium from cells not treated with Rottlerin. This effect of Rottlerin was seen in both the untreated and PMA treated cells. Since PGs are highly negatively charged molecules, the radioactively labelled macromolecules were subjected to Q-Sepharose chromatography. As shown in Figure 6C (upper panel), all the ^35^S-labelled material was PGs as it was eluted with 0.7 M NaCl. Rottlerin did not affect the position of the eluted material, indicating that it did not affect the sulphate density of the GAG-chains. The use of ^3^H-labelled glucosamine showed that the majority of the radioactive sugar is incorporated in the PGs (Fig. 6C, lower panel), but a small amount is also eluted at around 0.3 M NaCl which is most likely hyaluronan and non-PG glycoproteins. The fractions eluted from the Q-Sepharose column at the position of PGs (∼0.7 M NaCl) were pooled and divided in three parts. One was treated with cABC to degrade CS-chains, another was treated with NaOH to liberate the CS-chains from the core protein and the third part was untreated (control). These fractions were applied to a Superose 6 column. As seen in Figures 6(D) and 6(E), cABC totally degraded the GAG-chains to disaccharides showing that the radioactively labelled PGs were substituted with CS-chains. Furthermore, Rottlerin did not affect the type of GAG-chains synthesized. However, the size of the PGs synthesized by Rottlerin exposed cells was smaller than the PGs synthesized by the control cells. NaOH treatment of the material shows that also the size of the CS-chains was smaller in the Rottlerin treated material. Since free CS-chains with a molecular size less than 15 kDa would be expected to elute from the Q-Sepharose column at a lower NaCl concentration than CS-chains with equal charge density but with molecular size >20 kDa [54], [55], several experiments were performed with material from both the control and Rottlerin-exposed cells where the CSPG had been treated or not treated with NaOH. Figure 6F shows that the elution profile of the free CS-chains of the Rottlerin-treated and untreated materials superimposed on the corresponding intact PGs. In all cases, gel filtration on a Superose 6 column revealed that the CSPG from the Rottlerin exposed cells had a reduced size compared to the CSPG from the untreated cells, although the reduction in size varied from experiment to experiment. Thus, if there were some free CS-chains in the Rottlerin-treated material it was not possible to separate these chains from intact CSPGs by Q-Sepharose column chromatography at least during the conditions used in the present work. The reduced size of the CSPG from Rottlerin exposed cells compared to CSPG from the control cells was not detected when [^35^S]CSPG was subjected to SDS-PAGE (7.5% acrylamide) followed by autoradiography (Fig. 6F, upper panel). Both bands appeared at the top of the separating gel at a position that corresponded with the proMMP-9/CSPG complex seen in gelatin zymography. However when a 4–12% gradient gel was used, the reduced size of the CSPG from Rottlerin exposed cells was detected in spite of the smeared bands that appear due to the heterogenous size of the CS-chains (Fig. 6F, lower panel). In summary, Rottlerin treatment of the THP-1 cells was followed by a reduced synthesis of the proMMP-9/CSPG heteromer, both components in the heteromer as well as a reduced size of the CSPG and its CS chains.

### Rottlerin inhibits the activation of several PKC isoforms

**Figure 7:**
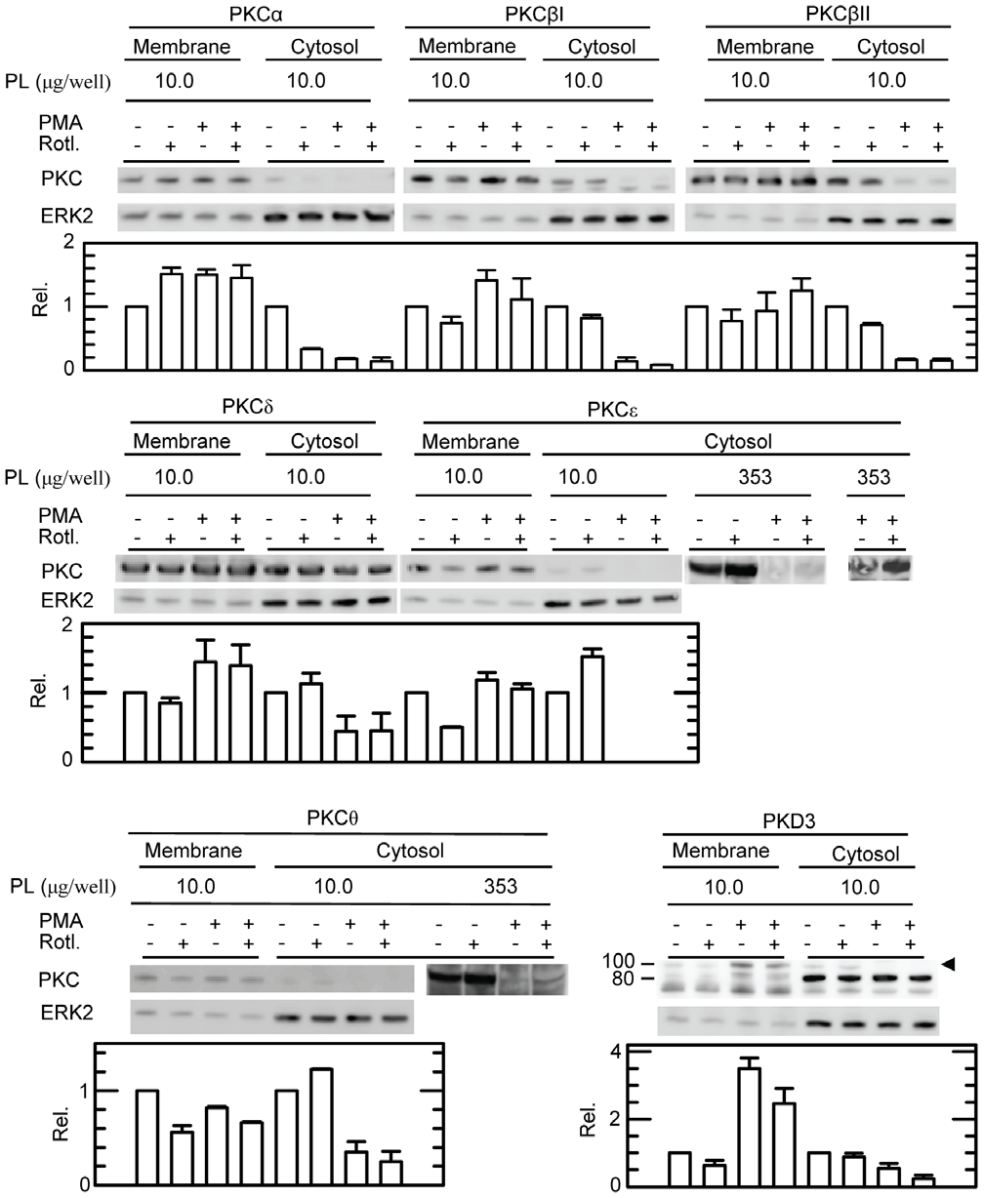
Effect of Rottlerin on cytosolic and plasma membrane bound PKC isoenzymes. Isolated cytosol and plasma membranes from untreated (−) or PMA and Rottlerin treated (+) THP-1 cells were subjected to Western blotting using PKC specific antibodies. The amount of total protein loaded (PL) to each well is shown. As an additional loading control, ERK2 was used. The diagram under each blot shows the relative amounts (mean ±s.d.) of the PKC isoenzymes in cytosol and membranes, where the results were normalised against the untreated cytosol and membrane controls. All results are based on equal protein loading and in all cases n = 2 except for PKC δ and υ (PKD3) where n = 3. The arrowhead shows the active PKD3 at 100 kDa, while the two bands with reduced molecular size may be truncated variants of PKD3. The position of the molecular mass markers at 100 and 80 kDa are shown at the left. In order to show the increased level of PKC ε in the cytosol from PMA treated cells (353 µg/well) in the presence of Rottlerin compared to the absence of this compound, a largely increased developmental exposure time of the membrane in the presence of the Luminol substrate was needed.

The inhibitory effect of Rottlerin on the biosynthesis of proMMP-9, CSPG and proMMP-9/CSPG heteromer may be due to inhibition of PKC activation or factors downstream to PKC. We have therefore determined the amount of PKC in the cytosol and the plasma membrane fraction of untreated and PMA treated THP-1 cells. To ensure equal loading, the total amount of cytosol and membrane proteins were determined as described in Materials and Methods. As shown in figure 7, PMA treatment of the cells resulted in decreased amounts of the classical and the novel PKCs in the cytosol as expected. PMA treatment resulted also in increased amounts of some PKCs to the plasma membranes. The presence of 5.0 µM Rottlerin resulted in a slight decrease in the amounts of the PKC isoenzymes δ, ε, θ and υ (PKD3; 100 kDa) bound to the plasma membranes in the PMA treated cells (Fig. 7). In the presence of Rottlerin, the PKC isoenzymes ε and θ showed a small increase in the cytosol of the PMA treated cells. In cells not exposed to PMA, the presence of Rottlerin resulted in a slightly increased amount of the PKC isoenzymes δ, ε and θ in the cytosol and a minor decrease in the plasma membrane (Fig. 7). MAPKAPK-2 is a mitogen-activated protein kinase expressed in THP-1 cells [56]. Because PMA also can stimulate the activity of MAPKAPK-2 [57], [58], and Rottlerin is a potent inhibitor of MAPKAPK-2 (IC_50_ = 5.4 µM; [59]), we examined whether PMA activated MAPKAPK-2 in THP-1 cells. No phosphorylation of MAPKAPK-2 at Thr-222 was detected in control cells or cell exposed to PMA, indicating that PMA did not activate MAPKAPK-2 in these cells (data not shown).

### Several factors that have an effect on either the MMP-9 or CSPG biosynthesis have no or little effect on the biosynthesis of the proMMP-9/CSPG heteromer

**Figure 8:**
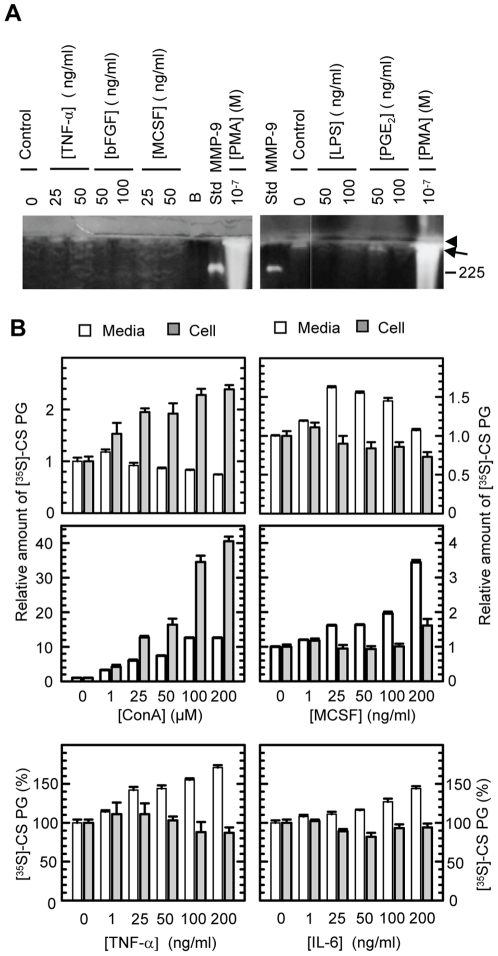
Effects of various compounds on the synthesis of proMMP-9/CSPG and CSPG. THP-1 cells were incubated in the absence or presence of the compounds shown in serum free medium for 72 h. In (**A**), the harvested medium was applied to Q-Sepharose chromatography and the presence of proMMP-9/CSPG was detected with gelatin zymography as described in Materials and Methods. Arrowhead shows the border between the separating and stacking gel and arrow shows the position of the 300 kDa proMMP-9/CSPG heteromer. The position of the pro-MMP-9 homodimer (225 kDa) is shown. **B**: Cells were incubated with [^35^S]sulphate. The harvested serum free medium (open bars) and the lysed cell preparations (grey bars) were passed over a G-50 Sepharose column in order to separate labelled macromolecules from free [^35^S]sulphate. The entire pass through fraction that contains the labelled macromolecules was then counted in a liquid scintillation spectrometer. All results (mean ± s.d) were normalized against the controls, i.e. the synthesis in absence of added compounds. In lower panel with ConA and MCSF, the results were in addition normalized against the number of viable cells. The results in (**B**) are from a typical experiment with four parallels.

In order to determine to what extent the heteromer biosynthesis could be induced by other biological factors than PMA, we investigated the effect of factors known to enhance MMP synthesis in various cell lines. Neither of the investigated factors did trigger a significant change in the synthesis of the proMMP-9/CSPG heteromer (Fig. 8A), although most of these compounds (TNF-α, M-CSF, IL-1α, IL-1β, IL-3, IL-6, LPS, PGE_2_ and ConA) caused an increase in the biosynthesis of proMMP-9 in a concentration dependent manner (data not shown). However, their stimulatory effect on proMMP-9 synthesis was 5–20 times less than the effect of PMA. No effect on the proMMP-9 synthesis was obtained with bFGF (data not shown).

ConA reduced, whereas TNF-α, M-CSF, IL-3, IL-6 slightly increased the [^35^S]CSPG released into the culture media (Fig. 8B, upper panel). However, when the released [^35^S]CSPG was normalized against the number of viable cells, a large increase in the CSPG was observed (Fig. 8B, lower panel). No change in released CSPG occurred when the cells were exposed to LPS, bFGF, IL-1α, IL-1β or PGE_2_ (data not shown). Only ConA increased cell associated [^35^S]CSPG, while the other compounds showed a decrease or no change in cell associated CSPG (Fig. 8B).

## Discussion

Previously we have shown that THP-1 cells produce a reduction sensitive complex between proMMP-9 and a CSPG core protein which may influence the tissue localization of the enzyme [38]. The formation of the proMMP-9/CSPG complex affects several properties of the enzyme, including activation and binding to collagen I and gelatin [39], [40]. Furthermore, we have shown that the CSPG core protein binds to both the hemopexin domain and the fibronectin-like repeats in the catalytic domain of the enzyme. The latter interaction prevented the enzyme from binding directly to collagen and gelatin [39], which is important for the gelatinases ability to degrade gelatin [22]–[31]. An increasing number of studies reveal that processing and degradation of protein and peptide substrates by various MMPs requires that the substrates not only interact with the active site but also regions outside the active site, referred to as noncatalytic sites or exosites [60]–[62]. In addition, the cellular and tissue localization of an individual MMP along with available substrates in its immediate environment may either prevent or promote disease progression [18], [63]–[65]. With respect to development of new drugs, it is therefore important to consider whether an enzyme or enzyme activity represent a target or anti-target. The development of new inhibitors against MMPs requires knowledge of specific exosites for different substrates and whether a given MMP forms complexes with other molecules that either block such exosites or create new exosites that may involve both the MMP and its binding partner. In the present study our aim was to determine how different factors, known to affect the synthesis of MMP-9 or CSPG, influence the formation of the proMMP-9/CSPG complex in THP-1 cells. Knowledge of how various factors affect the cellular synthesis of this complex and its individual components is important for the understanding of putative biological conditions promoting complex formation, as well as insight in the mechanism behind the formation of the complex.

In the present work it is shown that 10^−9^ to 10^−7^ M PMA strongly upregulates the THP-1 cells synthesis of the proMMP-9/CSPG heteromer and the proMMP-9 monomer and homodimer. However, these concentrations of PMA were also associated with some cell death. In a previous study on THP-1 cells [46] it was shown that PMA (10^−9^–10^−7^ M) induced the synthesis of MMP-9 in a time and concentration dependent manner similar to the observations in the present work. In contrast to our work, the authors did not observe that PMA induced differentiation was accompanied by any cell death. The reason for this discrepancy is not known. The observed cell death in the present work may suggest that the PMA induced increase in secreted proMMP-9/CSPG, proMMP-9 and CSPG is a result of release of intracellular stored molecules from dying cells. Several independent facts strongly argue against this possibility. (1) THP-1 cells and other monocytic cell lines do not store MMPs or other secretory proteins in granules. (2) Van Ranst and coworkers [46] showed that PMA treatment of THP-1 cells resulted in a steady state increase in MMP-9 mRNA for at least up to 48 h corresponding to the increase in secreted MMP-9. (3) In the present work it is shown that only 10% of the synthesized CSPG is associated with the cells, which confirms that CSPG is constitutively secreted in monocytes and macrophages [7]. (4) Gelatin zymography of lysed THP-1 cells shows a band at around 80 kDa that is approximately 100 times more intense than the 92 kDa MMP-9 band (data not shown). This 80 kDa band is not seen in the cell conditioned media. Therefore, it can be concluded that the effect of PMA on the amount of the proMMP-9/CSPG heteromer, proMMP-9 and CSPG secreted into the conditioned medium is due to an altered biosynthesis, which will be discussed below.

In this study we show that the proMMP-9/CSPG complex, proMMP-9 and CSPG increases with time in the cell conditioned media from both unstimulated and PMA stimulated THP-1 cells. Whether PMA was removed already after three hours, or if it was present during the entire incubation time of 72 h did not affect the synthesis of the proMMP-9/CSPG complex and proMMP-9. Previously it has been shown for some cell types that prolonged incubation with PMA desensitizes cells and results in a down regulation of activated PKCs [66]. The present work shows that prolonged incubation of THP-1 cells with PMA does not necessarily down regulate the PKCs and other factors involved in the induction of the cells synthesis of the proMMP-9/CSPG complex and proMMP-9. As extended exposure of cells to Rottlerin was toxic at concentrations above 5 µM, we could not incubate the cells in the presence of this compound for more than 8 hours in order to detect any effect on the activation of PKCs. However, this will give a reliable picture on the role of PKC isoenzymes in the synthesis of the proMMP-9/CSPG complex and proMMP-9 since it was sufficient to prime the cells for three hours with PMA in order to induce a prolonged increase in the cell synthesis of MMP-9 and its complex with CSPG.

As PMA induces activation of the classical and novel PKCs [51], [66], it can be assumed that one or several of these PKCs are involved in the PMA induced changes in the synthesis of proMMP-9/CSPG, proMMP-9, and CSPG by THP-1 cells. This assumption is supported by a previous work on THP-1 cells where it was shown that PMA induced synthesis of MMP-9 was not due to activation of PKA or increase in intracellular calcium [46]. Treatment of the THP-1 cells with the PKC inhibitors Gö6976 and Gö6983 reveals that the PMA induced synthesis of proMMP-9 and its complex with CSPG is independent of the PMA induced activation of PKC α, β or δ. Hence, the PMA induced synthesis is most likely due to the activation of one or several of the PKC isoforms ε, θ or υ (PKD3).

Rottlerin inhibited the synthesis of the proMMP-9/CSPG heteromer, proMMP-9 and CSPG in both the PMA treated and untreated THP-1 cells. Previously it has been shown that Rottlerin influences various cellular processes such as mitochondrial respiration [67], cell growth and viability [68], cell cycle distribution [68], generation of superoxide [69], signalling events and cytokine production [44] as well as macropinocytosis [70]. Rottlerin has been assumed to be a specific inhibitor of the two novel PKCs δ and θ [44], [52], [53], although this has been disputed [59], [67]. Activation of PKC is known to be associated with phosphorylation and transfer of the activated enzyme from the cytosol to various membrane fractions. The nature of the membrane fraction to which an activated PKC is translocated depends on the stimulatory conditions used [71]–[73]. Analysis of both the cytosol and the plasma membrane fraction should reveal whether or not Rottlerin inhibits the activation of a given PKC isoform. Inhibition should result in a decreased amount of the PKC in the plasma membrane fraction (if the activated form is bound to this membrane fraction) and an increased amount in the cytosol fraction. In the present study we show that under the conditions used, 5 µM Rottlerin inhibited the synthesis of the MMP-9/CSPG heteromer and proMMP-9. However, Rottlerin only slightly reduced the PMA induced activation of the PKC isoforms δ, θ and υ (PKD3) as well as the activated forms of PKC δ, θ and ε in the PMA untreated THP-1 cells. Thus it is not possible to conclude whether or to what extent these PKC isoenzymes are involved in the PMA induced synthesis of proMMP-9 and its complex with CSPG. Furthermore, PMA did not cause phosphorylation/activation of MAPKAPK-2, jeopardizing a role of MAPKAPK2 in PMA-triggered proMMP-9/CSPG synthesis. Therefore, the Rottlerin sensitivity of the synthesis of MMP-9 and its complex formation with CSPG in both the unstimulated and PMA stimulated cells may be due to Rottlerin acting on enzymes downstream the PKC isoforms δ, θ, ε and υ (PKD3) and/or on an alternative PMA induced PKC independent pathway. Indeed, Rottlerin has been demonstrated to inhibit the enzymatic activity of several other protein kinases [43] and PMA can enhance expression of several proteins in what was suggested to be in a PKC-independent pathway [74]–[77].

As for the synthesis of MMP-9, the synthesis of CSPG was reduced in the presence of Rottlerin. However, the synthesis of CSPG was reduced at concentrations of Rottlerin (0.5 to 1.0 µM) that did not affect the synthesis of the MMP-9/CSPG heteromer and proMMP-9. This suggests that the signalling pathway involved in the synthesis of CSPG is different from the one involved in the synthesis of the proMMP-9/CSPG heteromer and proMMP-9. Furthermore, at least a part of the Rottlerin induced reduction in CSPG (Figs. 6A,B) can be ascribed to the formation of PG core proteins containing shorter CS-chains compared to untreated controls. This was reflected in CSPG molecules with smaller molecular mass than the CSPGs produced in the absence of Rottlerin (Figs. 6D and E). Although the Rottlerin treated cells produced CSPGs with shorter CS-chains, there was no significant difference in charge density between the CSPGs from the untreated and Rottlerin treated cells. This was demonstrated by a similar elution profile from the Q-Sepharose column, where the CSPG from both the Rottlerin-treated and untreated cells were eluted at approximately 0.7 M NaCl (Fig. 6C). This suggests that Rottlerin inhibits one or several of the enzymes involved in CS-chain elongation and termination. In contrast to this, it appears that Rottlerin has no effect on the enzymes involved in the glycosylation of proMMP-9, as gelatin zymography of cell conditioned media did not reveal active bands with lower molecular mass than 92 kDa, but only less amounts of the 92 kDa form. There was no reduction in the amount of proMMP-9/CSPG heteromer formed in the presence of 1.0 µM Rottlerin compared to control (Fig. 5C). This suggests that Rottlerin only affected the size of CS-chains bound to the PG core proteins but had no effect on the amount or types of core proteins produced, or at least no changes in those PGs involved in the complex formation with proMMP-9.

PMA mimics diacylglycerol and in addition to the activation of the classical and novel PKCs, the Raf/MEK/ERK, JNK, p38/MAPKAPK2 and NFκB pathways can also be activated [78]-[81]. This results in the regulation of several genes, such as MMP-9, TNF-α, IL-1β and PGE_2_ [46], [50], [82], [83]. Several of the compounds used like LPS, ConA, M-CSF and IL-1β are known to activate at least one of the following regulators and transcription factors, TNF-α, NFκB, IRAK, ERK 1/2, p38, JNK, SAPK and PGE_2_ [44], [84]–[89]. Of the compounds used in the present work, PMA induced the largest increase in the synthesis of proMMP-9 and was the only agent that stimulated the synthesis of the proMMP-9/CSPG complex. This suggests that the pathways induced by TNF-α, M-CSF, IL-1α, IL-1β, IL-3, IL-6, LPS, PGE_2_ and ConA alone are not sufficient to increase the synthesis of the proMMP-9/CSPG complex in THP-1 cells. The difference in response between PMA and the other compounds mentioned above may be due to different types of CSPG core proteins synthesized and/or the synthesis of other molecules which prevent proMMP-9/CSPG complex formation. Several studies have shown a synergetic effect between various factors such as cytokines, growth factors and lipopolysaccharides in the regulation of MMPs [90]–[93]. Therefore, it cannot be excluded that several of the compounds used in the present study must act together in order to stimulate the synthesis of the proMMP-9/CSPG complex.

Our previous studies have revealed that the assembly of proMMP-9 and the core protein of one or several CSPGs must occur either inside the cells during the synthesis or in the extracellular microenvironment [38]. Recently we have found that the proMMP-9/CSPG complex can be reconstituted *in vitro*, and that the strong reduction sensitive interaction between the two molecules was not due to a disulfide bridge between the molecules (Malla N et al, unpublished). In all tissues, including tumours, there is a mixture of different cell types. If the proMMP-9/CSPG complex also can be formed in the extracellular environment, different cell types may contribute to the formation of this complex by producing one or both of the components of the heteromer. Thus the presence of a given CSPG in a tissue may affect the localization of proMMP-9. Since we previously showed the formation of the proMMP-9/CSPG complex affects several properties of the enzyme, including activation and binding to collagen I and gelatin [39], [40], the presence of a given CSPG in a tissue may also influence the activation and the proteolytic capacity of the enzyme. Based on our knowledge of MMP-9 and PGs in physiological and pathological processes, as well as the role of exosites in substrate cleavage [5], [12], [35], [94]–[101], one can not underestimate the putative biological role of an MMP-9/CSPG complex.
